# Prenatal diagnosis of fetuses with ultrasound anomalies by whole-exome sequencing in Luoyang city, China

**DOI:** 10.3389/fgene.2023.1301439

**Published:** 2024-01-22

**Authors:** Yanan Wang, Fan Yin, Yuqiong Chai, Jiapei Jin, Pai Zhang, Qianqian Tan, Zhigang Chen

**Affiliations:** ^1^ Department of Genetics and Prenatal Diagnosis, Luoyang Maternal and Child Health Hospital, Luoyang, China; ^2^ Puluo (Wuhan) Medical Biotechnology Co., LTD., Wuhan, China

**Keywords:** prenatal diagnosis, ultrasonographic anomalies, WES, diagnostic yield, pregnancy outcomes

## Abstract

**Background:** There is a great obstacle in prenatal diagnosis of fetal anomalies due to their considerable genetic and clinical heterogeneity. Whole-exome sequencing (WES) has been confirmed as a successful option for genetic diagnosis in pediatrics, but its clinical utility for prenatal diagnosis remains to be limited.

**Methods:** A total of 60 fetuses with abnormal ultrasound findings underwent karyotyping or chromosomal microarray analysis (CMA), and those with negative results were further subjected to WES. The identified variants were classified as pathogenic or likely pathogenic (P/LP) and the variant of uncertain significance (VUS). Pregnancy outcomes were obtained through a telephone follow-up.

**Results:** Twelve (20%, 12/60) fetuses were diagnosed to have chromosomal abnormalities using karyotyping or CMA. Of the remaining 48 cases that underwent WES, P/LP variants were identified in 14 cases (29.2%), giving an additional diagnostic yield of 23.3% (14/60). The most frequently affected organ referred for prenatal WES was the head or neck system (40%), followed by the skeletal system (39.1%). In terms of pathogenic genes, *FGFR3* was the most common diagnostic gene in this cohort. For the first time, we discovered five P/LP variants involved in *SEC24D*, *FIG4*, *CTNNA3*, *EPG5*, and *PKD2*. In addition, we identified three VUSes that had been reported previously. Outcomes of pregnancy were available for 54 cases, of which 24 cases were terminated.

**Conclusion:** The results confirmed that WES is a powerful tool in prenatal diagnosis, especially for fetuses with ultrasonographic anomalies that cannot be diagnosed using conventional prenatal methods. Additionally, newly identified variants will expand the phenotypic spectrum of monogenic disorders and greatly enrich the prenatal diagnostic database.

## 1 Introduction

Congenital anomalies, ranging from relatively minor to severe multi-system anomalies, affect approximately 3 of 100 live births and are responsible for 21% of perinatal deaths (Ely Driscoll, 2020). Identification of the cause of congenital anomalies is important for genetic counseling and clinical management. Ultrasonography, which provides information on the presence or absence of bones, fetal size, shape, and position, is routinely used in prenatal care (Dhamankar et al., 2020; Macedo et al., 2022). However, ultrasound has difficulties in clearly distinguishing between different types of anomalies, resulting in limited prenatal phenotype information.

Typically, fetuses with ultrasonographic anomalies can be associated with all types of genetic variation, such as chromosome aneuploidies, copy number variations, and single-base mutation. Generally, G-band karyotyping that detects chromosome aneuploidies and unbalanced rearrangements (>5–10 Mb) is recommended as the first-line test for prenatal diagnosis with a diagnostic yield of 32% in fetuses with a structural abnormality. In addition, chromosomal microarray analysis (CMA) that identifies smaller microdeletions and duplications is also offered to improve diagnosis by up to 6% over G-banded karyotyping (Wapner et al., 2012). However, only a proportion of fetal anomalies have clear molecular pathogenesis, and the cause of a large number of fetuses with abnormal ultrasound remains elusive, resulting in challenges in prenatal counseling.

Next-generation sequencing (NGS) technology has been widely used for identification of causative genes in various genetic disorders, including intellectual disability (Vrijenhoek et al., 2018), inherited peripheral neuropathies (Hartley et al., 2018), and epilepsy (Symondsand McTague, 2020). Most recently, a clinical guideline of the American College of Medical Genetics and Genomics (ACMG) highly recommended exome and genome sequencing as a first- or second-tier test for patients with developmental delay or intellectual disability (Manickam et al., 2021). Whole-exome sequencing (WES) plays an important role in postnatal diagnosis, while only recently has WES obtained importance in prenatal diagnosis. Carss et al. (2014), who first introduced the WES strategy into prenatal diagnosis in a cohort of 30 non-aneuploid fetuses, identified 35 *de novo* single-nucleotide variants, small indels, deletions, or duplications. Causative variants were identified in three out of 30 cases with a diagnostic yield of 10%. Several prior studies have examined the use of prenatal WES, where either karyotype analysis or CMA yielded negative results. These studies reported diagnostic yields ranging from 9.1% to 45.9% (Leung et al., 2018; Sparks et al., 2020; Vora et al., 2020; Huang et al., 2023). These findings highlight the significant contribution of WES toward improving the prenatal diagnostic rates.

Despite the numerous advances in WES technology, conclusions drawn from the majority of pilot studies are biased by small cohorts (Cao et al., 2022; Yang et al., 2022) or are limited to highly selected cases or other certain conditions (Liu et al., 2019; Lei et al., 2022). Further pilot studies involving different disorders, conditions, sample types, and analytic methods are necessary to expand our understanding in the application of WES on prenatal diagnosis and to establish the diagnostic yield of WES for all kinds of prenatal disorders.

Here, we present 60 prenatal cases with various ultrasonographic anomalies. All pregnant women underwent invasive testing, either via karyotyping or CMA or both. Cases with negative results of karyotyping or CMA tests were further subjected to WES for causative variant identification, with the aim of discovering more pathogenic mutations and clarifying the utility of WES in prenatal diagnosis.

## 2 Materials and methods

### 2.1 Study cohort

**FIGURE 1:**
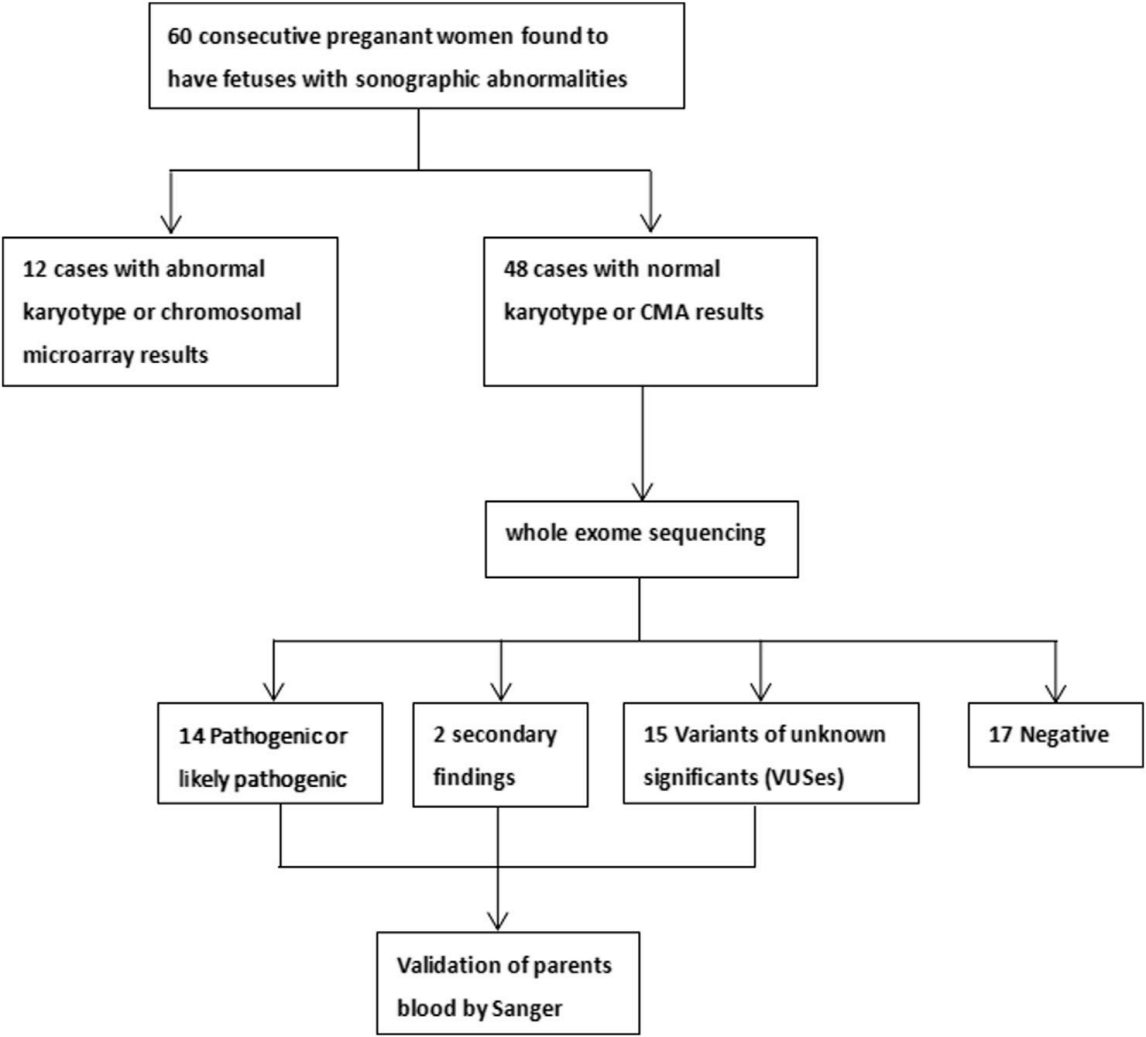
Study profile.

This study was granted by the Institutional Review Board of the Ethics Committee at the Luoyang Maternal and Child Health Hospital (approval number LYFY-YCCZ-2023006). Pregnant women were recruited at our hospital between November 2019 and December 2022, from which 60 pregnancies with ultrasonographic anomalies that underwent invasive diagnosis were selected for further investigation. All pregnant women and their partners signed written informed consent for invasive procedures, testing, and participation. Phenotypic information and disease classification were provided by the clinicians, according to the ultrasonographic results based on the Human Phenotype Ontology (HPO) and the Online Mendelian Inheritance in Man (OMIM) databases. If a case had multiple-organ abnormalities, then each abnormality was counted separately. The study profile is shown in Figure 1.

### 2.2 Chromosome karyotyping and chromosomal microarray analysis

Amniotic fluid samples (10 mL) were collected from pregnant women and subjected to conventional G-band karyotyping, according to standard operation procedures as previously described (Shi et al., 2019). For CMA analysis, genomic DNA was extracted from amniotic fluid using a QIAamp DNA Mini Kit (QIAGEN Inc., Valencia, CA, United States), followed by amplification, labeling, and hybridization, and then, a CMA test was performed using a 750K microarray chip (Affymetrix Inc., United States), according to the manufacturer’s protocol. The classification of pathogenic copy number variations (CNVs) was determined according to international guidelines of the ACMG.

### 2.3 Whole-exome sequencing

Fetuses with normal karyotype analysis or CMA results were further subjected to WES for causative variant identification. As previously described (Lippa et al., 2021), WES procedures including library construction, exome capture, and high-throughput sequencing were performed. In brief, 150 ng of genomic DNA was fragmented (250–300 bp), and then, library preparation, including end repair, A-tailing, adapter ligation, and PCR amplification, was conducted using the IDT xGen Exome Research Panel v1.0 (Integrated DNA Technologies, San Diego, United States), according to the manufacturer’s protocols. After getting captured by VCRome version 2.1, all exome libraries were pooled and sequenced using the NovaSeq6000 PE150 platform (Illumina, San Diego, United States), with a target sequencing coverage of 100× and with over 95% of the exonic positions with the depth of coverage >20×.

The QC of the paired-end reads was assessed using FastQC, and raw sequence data were post-progressed on-site using open-source software. After filtering using SAMtools, the sequencing reads were aligned to the human genome reference sequence (hg19/GRCh37) using the Burrows–Wheeler Aligner (BMA, version 0.59). GATK HaplotypeCaller v3.6 was used to detect single-nucleotide variants (SNVs) and indels (Van der Auwera et al., 2013), and then, the variants were annotated using ANNOVAR (Monday, 8 June 2020). Small chromosomal CNVs and single-gene CNVs were analyzed using CNVkit and annotated using AnnotSV. In the present study, the clinical significance of identified variants was classified into the following three categories, namely, pathogenic (P), likely pathogenic (LP), and the variant of uncertain significance (VUS), according to the ACMG guidelines (Green et al., 2013), based on the allele frequency, family segregation, compatibility with the phenotype, *in silico* prediction, relevant disease databases, and the literature. The allele frequency ≤0.07% in the gnomAD database was considered a variant of clinical significance. Moreover, the Human Gene Mutation Database (HGMD) was used to assess whether the variant identified in the present study had been previously reported.

### 2.4 Sanger sequencing

All variants considered to be causative for the observed fetal phenotype were validated by Sanger sequencing using both fetal amniotic fluid DNA and parental peripheral blood samples. Further clinical tests were performed, and additional family history information was collected to facilitate interpretation of the Sanger sequencing results.

### 2.5 The pregnancy outcome

Pregnancy outcomes were collected through follow-up telephone calls until August 2023, including the condition and phenotype of live-born infants. The phenotype of the parents was also recorded.

## 3 Results

### 3.1 Detection of fetal chromosomal abnormalities

A total of 60 pregnant women of Han ethnicity with ultrasonographic anomalies were referred to the Luoyang Maternal and Child Health Hospital. Supplementary Table S1 displays the demographics of the pregnant women, revealing that they had a median maternal age of 29.5 ± 4.3 years (range 21–39) and a median gestational age of 23.4 ± 4.9 weeks (range 12–33). Invasive procedures were conducted for all subjects, of which 23 cases received CMA and the other 37 cases received both G-band karyotyping and CMA. Totally, 12 out of 60 (20%) cases were found to have chromosomal abnormalities. One case was a fetus with Down’s syndrome, and another had mosaic aneuploidy. The remaining 10 fetuses with abnormal copy number variants ranging from 150 kb to 4.3 Mb were mainly involved in Duchenne muscular dystrophy, intellectual disability, X-linked 93, glass syndrome, hereditary hearing loss, and renal hypoplasia. All the copy number variants were classified to be pathogenic or likely pathogenic or the variant of uncertain significance, according to ACMG (Table 1).

**TABLE 1:** Characteristics of fetuses with copy number variations detected by chromosomal microarray analysis.

Case	Main ultrasound finding	Microarray result (ISCN)	Size	Inheritance or *de novo*	Associated disorder^b^	ClinGen Dosage ID (score)	ACMG classification (Green et al., 2013)	Karyotyping	Pregnancy outcome^a^
**1**	Renal dysplasia and macrocephaly	arr[GRCh37]9p22.3 (14803711_14907432)×1	103.7 kb	*De novo*	Trigonocephaly 2	ISCA-23907 (HI 0)	Likely pathogenic (4A)	NA	TOP
						PMID:21931569			
**2**	Short femur and seroperitoneum	arr[GRCh37]18p11.21-q11.2 (15260001_19560000)×1	4.3 Mb	Unknown	Hereditary hearing loss	PMID:29100090	Pathogenic (2A)	NA	TOP
					Renal hypoplasia	PMID:32378186 ^c^			
**3**	Hydronephrosis and ventricular septal defect	arr[GRCh37]2q33.1 (199379852_201279851)×1	1.9 Mb	*De novo*	Glass syndrome	ISCA-31904 (HI 3)	Pathogenic (2A)	46,XX	Live birth with groan breathing
**4**	Absent fetal nasal bone and ventricular septal defect	NA	NA	NA	Down’s syndrome	NA	Pathogenic (2A)	47,XY,+21	TOP
**5**	Polyhydramnios, short femur, and abnormality of the gallbladder	arr[GRCh37]16p11.2 (28760001_29010000)×1	250 Kb	Unknown	Chromosome 16p11.2 deletion syndrome	ISCA-37486 (HI 3)	Pathogenic (2A)	NA	TOP
**6**	Increased nuchal translucency	arr[GRCh37]Xq21.1 (80060001_80210000)×0	150 Kb	Inherited maternally	Intellectual disability and X-linked 93	ISCA-16544 (HI 3)	Likely pathogenic (2C-1)	46,XY	Live birth without any abnormality
7	Duodenal ileus	arr[GRCh37]16p11.2 (29560001_30210000)×1	650 kb	Unknown	Chromosome 16p11.2 deletion syndrome	ISCA-37400 (HI 3)	Pathogenic (2A)	46,XX	TOP
8	Imperforate atrioventricular valve and pulmonary artery atresia	arr[GRCh37]22q11.2 (22250005_22550004)×3	300 kb	Unknown	22q11.2 deletion syndrome	NA	VUS	NA	TOP
9	Partial absence of the cerebellar vermis	NA	NA	NA	Mosaic trisomy 2	NA	Pathogenic (2A)	47,XY,+2[11]/46,XY[46]	Live birth without abnormality
10	Short femur	arr[GRCh37]Xp21.1 (31860001_32110000)×1	250 kb	Unknown	Duchenne muscular dystrophy	ISCA-6635 (HI 3)	Pathogenic (PVS + PS4+PM2__Supporting)	46,XX	Live birth without abnormality
11	Increased nuchal translucency	arr[GRCh37]16p13.11 (15510001_16360000)×3	850 kb	Inherited paternally	16p13.11 recurrent region	ISCA-37415 (TS 2)	Pathogenic (2A)	46,XY	Live birth without abnormality
12	Short fetal length and bilateral renal echo enhancement	arr[GRCh37]17q12 (34800001_36250000)×1	1.45 Mb	Unknown	Chromosome 17q12 deletion syndrome	ISCA-37432 (HI 3)	Pathogenic (2A)	NA	TOP

“TOP” denotes the termination of pregnancy.

“NA” denotes not applicable.

a After termination, the fetal samples were not used for further diagnosis.

b Associated disorder was determined according to OMIM.

c LOF, variants of the involved gene GRE1BL, have been reported to be pathogenic (PMID: 29100090, PMID: 32378186, ClinVar ID: 2445432, et al.). In addition, two or more haploinsufficiency predictors suggest it is haploinsufficient.

### 3.2 Positive diagnostic results identified by WES

After exclusion of 12 fetuses with chromosome abnormalities, the remaining 48 amniotic fluid DNA samples were then subjected to WES. As shown in Table 2, 14 (29.2%) cases were found to be harboring P/LP variants. Accordingly, WES increased the diagnostic yield by further 14 cases (23.3%) above routine genetic testing. In total, 13 P/LP variants were identified in 14 fetuses corresponding to 11 unique genes. Seven (50%, 7/14) fetuses had a *de novo* mutation, with *FGFR3* being the most frequent diagnostic gene carrying the same mutation c.1138G>A (p.G380R) that was identified in four cases. Four (28.6%, 4/14) fetuses inherited the mutation from parents who were heterozygous carriers in an autosomal recessive form, including compound heterozygous *SEC24D*, *EPG5*, and *FIG4*, as well as a homozygous *P3H1*. The remaining three fetuses (21.4%, 3/14) inherited the mutation in an autosomal dominant way from one of their parents (*HNF1B*, *CRYAA*, and *CTNNA3*). Of the 13 P/LP variants, we have identified five variants that are not currently included in the HGMD. These variants included a *de novo* missense mutation in the *PKD2* gene in fetuses suspected of causing polycystic kidney dysplasia; a maternally transmitted *FIG4* canonical splice variant; a maternally transmitted *EPG5* missense variant linked to vici syndrome; a maternally transmitted *CTNNA3* canonical splice variant associated with arrhythmogenic right ventricular dysplasia; and a maternally transmitted *SEC24D* canonical splice variant, which may lead to Cole–Carpenter syndrome 2.

**TABLE 2:** Summary of pathogenic and likely pathogenic mutations revealed by whole-exome sequencing.

Case	Main ultrasound finding	Gene	Associated disorder^c^	Alteration	Variant type	HGMD inclusion	Function prediction	Inheritance/zygosity	ACMG classification (Green et al., 2013)	Pregnancy outcome^b^
							REVEL/spliceAI effect			
**13**	Lateral femoral bowing	*SEC24D*	Cole–Carpenter syndrome 2	NM_001318066.2 c.2241 + 1G>T	Canonical splice	-	NA/Yes	Inherited/compound heterozygous	**Likely pathogenic** (PVS1+PM2_Supporting)	Born with large fontanelles
				NM_001318066.2 c.941G>A (p.R314H)	Missense	CM1617705	0.685/NA		**Likely pathogenic** (PM3_Strong + PM2_Supporting + PP3)	
**14**	Spot in the left ventricle and bilateral femoral bowing	*P3H1*	Osteogenesis imperfecta	NM_001243246.2 c.2041C>T (p.R681^a^)	Nonsense	CM086851	NA/NA	Inherited/homozygous	**Likely pathogenic** (PVS_Moderate + PS3_Supporting + PM2_Supporting + PM3)	TOP
**15**	Bilateral talipes equinovarus	*NALCN*	Congenital contractures of the limbs, face, hypotonia, and developmental delay	NM_001350748.2 c.1733A>G (p.Y578C)	Missense	CM176873	0.976/NA	*De novo*/heterozygous	**Pathogenic** (PS2_Very strong + PM2_Supporting, PP3)	Born with bilateral talipes equinovarus combined with curved fingers
**16**	Bilateral hyperechogenic kidneys	*HNF1B* ^a^	Renal cysts and diabetes syndrome	NM_000458.4 c.809 + 1G>A	Canonical splice	CS104578	NA/Yes	Inherited paternally/heterozygous	**Likely pathogenic** (PVS + PM2_Supporting)	Born with bilateral cortical cysts
**17**	Enhanced parenchymal echo in both the kidneys	*PKD2*	Polycystic kidney dysplasia	NM_000297.4 c.1034A>G (p.Y345C)	Missense	-	0.855/NA	*De novo*/heterozygous	**Likely pathogenic** (PM1+PM2_Supporting + PM6+PP3)	Lost to follow-up
**18**	Lens opacity	*CRYAA* ^a^	Congenital cataract	NM_000394.4 c.160C>T (p.R54C)	Missense	CN076130	0.824/NA	Inherited paternally/heterozygous	**Likely pathogenic** (PS3+PS4__Supporting + PM2_Supporting + PP3)	Lost to follow-up
**19**	Short femur and small belly circumference	*FGFR3*	Achondroplasia	NM_000142.5 c.1138G>A (p.G380R)	Missense	CM940785	0.696/NA	*De novo*/heterozygous	**Pathogenic** ((PS2+PS3_Supporting + PM1+PM5+PM2_Supporting))	TOP
**20**	Short femur, short humerus, and polyhydramnios	*FGFR3*	Achondroplasia	NM_000142.5 c.1138G>A (p.G380R)	Missense	CM940785	0.696/NA	*De novo*/heterozygous	**Pathogenic** (PS2+PS3_Supporting + PM1+PM5+PM2_Supporting)	TOP
**21**	Skeletal dysplasia	*FGFR3*	Achondroplasia	NM_000142.5 c.1138G>A (p.G380R)	Missense	CM940785	0.696/NA	*De novo*/heterozygous	**Pathogenic** (PS2+PS3_Supporting + PM1+PM5+PM2_Supporting)	TOP
**22**	Skeletal dysplasia	*FGFR3*	Achondroplasia	NM_000142.5 c.1138G>A (p.G380R)	Missense	CM940785	0.696/NA	*De novo*/heterozygous	**Pathogenic** (PS2+PS3_Supporting + PM1+PM5+PM2_Supporting)	TOP
**23**	Double aortic arch and patent ductus arteriosus	*CTNNA* ^a^	Arrhythmogenic right ventricular dysplasia, 13	NM_001127384.3 c.1281 + 1G>A	Canonical splice	-	NA/Yes	Inherited maternally/heterozygous	**Likely pathogenic** (PVS + PM2_Supporting)	Live birth without abnormality
**24**	Short femur and femoral bowing	*COL1A2*	Osteogenesis imperfecta	NM_000089.4 c.821G>A (p.G274D)	Missense	CM011295	0.983/NA	*De novo*/heterozygous	**Likely pathogenic** (PM1+PM6+PS4_Moderate + PM2_Supporting + PP3)	TOP
**25**	Leukodystrophy	*EPG5*	Vici syndrome	NM_020964.3 c.7333C>T (p.R2445^a^)	Nonsense	CM163403	NA/NA	Inherited/compound heterozygous	**Likely pathogenic** (PVS + PM3_Supporting + PM2_Supporting)	TOP
				NM_020964.3 c.5714G>A (p.R1905Q)	Missense	-	0.657/NA		**Likely pathogenic** (PM1+PM3+PM2_Supporting + PP3)	
**26**	Dilated ventricle and fetal pyelectasis	*FIG4*	Yunis–Varon syndrome	c.2376 + 1G>T	Canonical splice	-	NA/Yes	Inherited/compound heterozygous	**Likely pathogenic** (PVS + PM2_Supporting)	TOP
				NM_014845.6 c.350C>T (p.A117V)	Missense	CI2215100	0.664/NA		VUS (PM2_Supporting + PM3+PP3)	

“-” denotes the variant that was not included in the HGMD.

^a^ denotes the mutation was inherited from the parent, and the disease displays incomplete penetrance or a highly variable phenotype.

^b^ After termination, the fetal samples were not used for further diagnosis.

“TOP” denotes termination of pregnancy.

^c^ Associated disorder was determined according to OMIM.

“NA” denotes not applicable.

“Yes” denotes has effect.

“PM2_Supporting” allele frequency <0.07%.

### 3.3 Secondary findings and variants of uncertain significance (VUSes)

In addition to P/LP variants, we also identified secondary findings associated with *GJB2, DUOX2*, and *TTN* genes in two cases (Supplementary Table S2). In addition, we detected 22 VUSes among 15 cases, three of which have been previously reported at least once (Supplementary Table S3). The remaining 17 cases were WES-negative; neither chromosomal CNVs nor single gene CNVs were identified in these negative cases (Supplementary Table S4).

### 3.4 Diagnostic rates of WES among ultrasonographic anomalies

**FIGURE 2:**
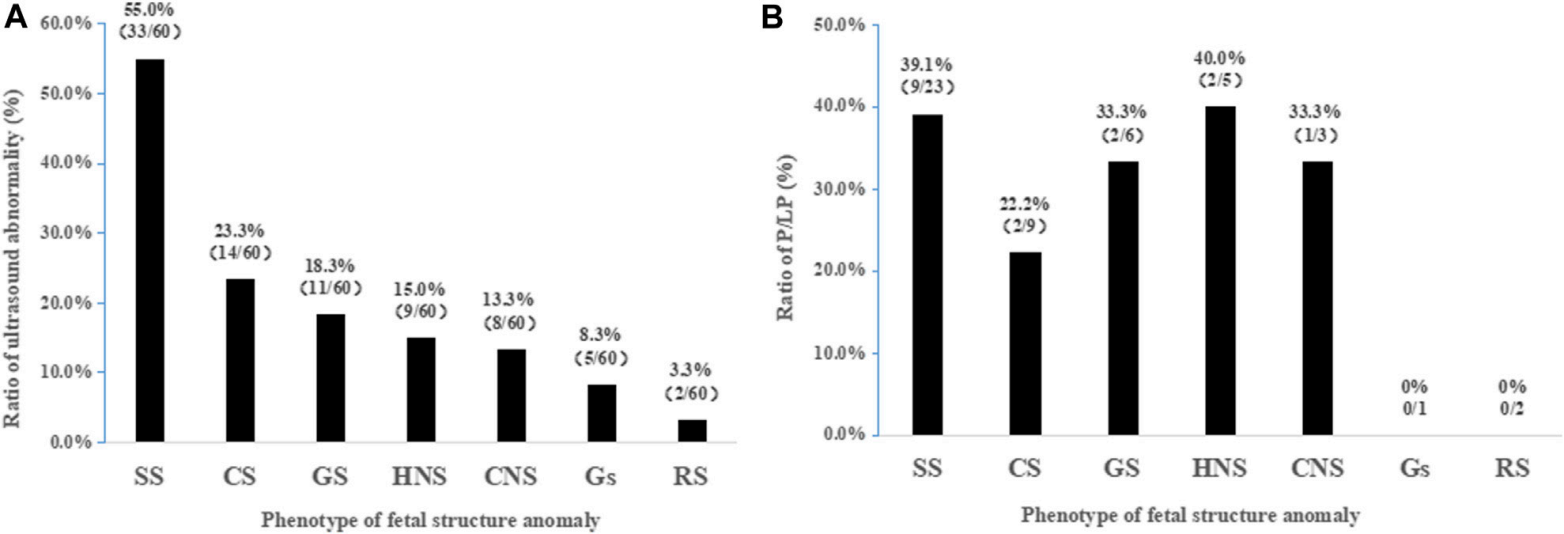
Frequency of diagnostic rates based on malformation classification. **(A)** Frequency of anomalies detected by ultrasound based on a total of 60 cases. **(B)** Diagnostic rates of WES among ultrasonographic anomalies. **SS**, skeletal system; **CS**, cardiovascular system; **GS**, genitourinary system; **HNS**, head or neck system; **CNS**, central nervous system; **GS**, gastrointestinal system; **RS**, respiratory system; P/LP, pathogenic or likely pathogenic.

We classified the fetal anomaly into seven categories, according to the systems involved, including skeletal, cardiovascular, genitourinary, head or neck, central nervous, gastrointestinal, and respiratory systems. Among the different malformation categories, the skeletal system (55.0%, 33/60) showed the most frequent findings in this cohort by ultrasonic testing, followed by cardiovascular (23.3%), genitourinary (18.3%), and head or neck systems (15.0%) (Figure 2A). The results in Figure 2B showed that diagnostic yield varied between different categories; the highest rate was found in the fetus with head or neck abnormalities (40%, 2/5) and skeletal abnormalities (39.1%, 9/23). In addition, the diagnostic rates in cardiovascular, genitourinary, and central nervous systems were 22.2% (2/9), 33.3% (2/6), and 33.3% (1/3), respectively. However, the sample size was too small that the test failed to detect meaningful differences in diagnostic rates for various systemic diseases.

### 3.5 Pregnancy outcomes

Pregnancy outcomes were available in 54 of 60 (90.0%) cases, and the remaining six cases were lost to follow-up. Twenty-four of 54 (44.4%) pregnancy women preferred termination, including seven fetuses identified with pathogenic copy number variations, eight fetuses with P/LP mutations, one fetus with secondary findings, four fetuses with VUSes, and four fetuses with negative results (Supplementary Table S4). It is noteworthy that the fetal samples were not subjected to further diagnosis post-termination. Of the 30 pregnancy women who chose to continue gestation, adverse pregnancy was observed in eight fetuses. In Table 1, the fetus in case 3 that was diagnosed to have glass syndrome was born with groan breathing. Three fetuses carrying the P/LP variant were born with abnormalities (Table 2), especially case 13, who was suspected to have Cole–Carpenter syndrome 2, was born with large fontanelles. Case 16 showed bilateral cortical cysts at birth, and case 15 presented with bilateral talipes equinovarus combined with curved fingers. The results were consistent with both the ultrasonographic findings and phenotype of the *NALCN* mutation. Interestingly, bilateral talipes equinovarus after birth was also observed in case 42 who was ultimately classified as having a VUS of the *SOX9* gene mutation (Supplementary Table S3). Additionally, two fetuses carrying VUS were born with abnormalities including a live birth with polymicrogyria in case 36 and moderate postnatal growth restriction in case 37. Another fetus with negative results was born with slight postnatal growth restriction in case 56. Of the 17 WES negative cases, 10 (58.8%) fetuses were live births with no abnormalities and one (5.9%) with an abnormality (Supplementary Table S4).

## 4 Discussion

In this study, WES was used to examine fetuses with abnormal ultrasound findings but could not be diagnosed by routine testing. Our findings demonstrated that WES increased the diagnostic yield in fetuses with abnormal sonographic findings by 23.3%, following negative results obtained through karyotype and CMA testing. These results are consistent with previously published series on published prenatal WES, which have reported diagnostic yields ranging from 15% to 25% (Vora et al., 2017; Vora et al., 2020; Lei et al., 2021). Our results further confirmed the potential of WES as a promising tool for extended prenatal diagnosis. The decreasing turnaround time and increasing accessibility, as well as the falling cost of next-generation sequencing will make it more feasible to use the WES strategy clinically. This will remarkably promote parental counseling and pregnancy management. Nevertheless, conventional testing cannot be replaced by WES due to limitations of WES in detecting large insertions/deletions, chromosomal rearrangements, and mutations in regulatory regions (Alotibi et al., 2023). Karyotype analysis or CMA in combination with WES would be a valuable strategy in prenatal testing.

Among seven categories in this cohort, skeletal anomalies and head or neck anomalies were the top two positive predictors of monogenic disorder. Here, we provided a diagnostic rate by WES of 39.1% of fetuses with abnormalities in the skeletal system, which was comparable to previously published prenatal WES studies with 30.4% (Fu et al., 2022) and 32.7% (Kucińska-Chahwan et al., 2022). However, a significantly higher rate of 75% has been reported elsewhere (Liu et al., 2019; Yang et al., 2019). Skeletal anomalies are a heterogeneous group of disorders affecting bones, cartilage, tendons, and joints (Köhler et al., 2017); a highly heterogeneous phenotype may contribute to the differences in detection rates between these studies. In addition, differences in inclusion criteria, sample size, or the definition of a positive result might also be involved. In any case, a higher diagnostic yield would be obtained from these malformations by WES with optimal cost-effectiveness. Therefore, WES is strongly recommended for fetuses with abnormal ultrasound findings of skeletal anomalies and head or neck anomalies.

With regard to pathogenic genes identified by WES, *FGFR3* was one of the most frequently diagnosed genes in the current study that is consistent with previous findings (Han et al., 2020; Zhang et al., 2021). However, different variants of *FGFR3* were responsible for different disorders with various phenotypes and prognoses that were unable to distinguish from fetal sonographic indicators (Yang et al., 2019). For instance, *FGFR3*: c.1138G>A (p.G380R) mutation accounted for 90% of the condition in achondroplasia patients (Bellus et al., 1995), while the *FGFR3*: c.1620C>A (p.N540K) mutation occurred in 70% of individuals with hypochondroplasia (Bellus et al., 2000). Therefore, WES is of importance for diagnosis and genetic counseling to fetuses with variations in identical genes.

A homozygous nonsense variation c.2041C>T (p.R681*) in *P3H1* was identified in case 14 with bilateral femoral bowing. The parent denied consanguinity, and the fetus was eventually terminated because this mutation is implicated in osteogenesis imperfecta. The same mutation has been reported in a British family, in which the fetus exhibited abnormal ultrasound findings of short, thick bowed femurs, evidence of fractures, short humerus, and abnormal shape (Chandler et al., 2018). In a consanguineous family of Arabic descent, this mutation was identified in a fetus with severe limbs and chest deformities (Baldridge et al., 2008). All these lines of evidence suggested that the homozygous non-sense variation c.2041C>T (p.R681*) in *P3H1* may be a common variant among different races and usually result in severe fetal malformation.

The *PKD2* gene, encoding polycystin-2, is associated with polycystic kidney disease, and the clinical symptoms usually do not appear until adulthood, but the disease starts *in utero* (Janssens et al., 2021). In one study, the author reported on a family carrying a mutation in the *PKD2* gene perinatal death due to polycystic kidney disease occurred in the mother’s second and third pregnancies (Bergmann et al., 2008). In case 17, a mutation in the *PKD2* gene was identified in a fetus with enhanced parenchymal echo in both the kidneys. This case was lost to follow-up, while the genetic result suggested a high risk of polycystic kidney dysplasia in adulthood; therefore, advanced interfere treatment is a matter of great urgency.

In case 23, the LP variant of *CTNNA3* was identified in the fetus with patent ductus arteriosus, but the transmitting mother was not an affected individual. This is largely due to the incomplete penetrance of this mutation in different affected individuals (van Hengel et al., 2013). A maternally transmitted canonical splice variant c.809 + 1G>A in *HNF1B* was identified in case 16 with bilateral renal cortical cysts after birth, while the same mutation inherited from the father was also reported in a previous study, in which the fetus was born with a unilateral ureteropelvic junction and renal failure, apart from bilateral renal cortical cysts (Heidet et al., 2010). These findings indicated that the severity of the renal disease resulting from the *HNF1B* mutation was highly variable. In case 18, the fetus inherited a heterozygous missense variation c.160C>T (p.R54C) in the *CRYAA* gene from the father, and the parents refused to disclose any information about the newborn. Meanwhile, the father reported no notable ocular disease. Khan et al. (2007) reported that three carriers of c.160C>T (p.R54C) in the *CRYAA* gene were asymptomatic but had similar bilateral discrete punctuate lenticular opacities evident by a careful slit lamp examination (Khan et al., 2007). Thus, in our study, fetal case 18 was highly suspected of congenital cataract, and we recommended further ophthalmic examination to the transmitted father, but it was not adopted. A sustained follow-up is necessary. To the best of our knowledge, five pathogenic or likely pathogenic variants implicated in *SEC24D*, *FIG4*, *CTNNA3*, *EPG5*, and *PKD2* (Table 2) had never been reported prenatally. These newly identified variants demonstrate how prenatal WES expanded the phenotypic spectrum of monogenic disorders in prenatal diagnosis. In a word, regarding the 14 cases with pathogenic or likely pathogenic genes (Table 2), the pregnancy outcomes further supported the main ultrasound findings only in two cases (cases 15 and 16). However, the concordance of the remaining cases was indeterminate, either because of pregnancy termination or loss to follow-up.

The annotation of VUS in the prenatal setting has been debated for years, and it is essential that VUS results are properly explained to avert potential misuse (Cornthwaite et al., 2022). Here, we identified 22 VUSes (Supplementary Table S3), of which three variants (JAG1:c.1136C>T(p.S379F), NBAS:c.6124A>G (p.M2042V), and DYNC2H1:c.7409C>T (p.A2470V)) had been reported previously at least once. We firmly believe that with the promotion of WES in prenatal diagnosis, more VUSes will be identified and reclassified, allowing a more accurate diagnosis will be possible with integrated genotype–phenotype information.

Although WES has many advantages over the traditional test in prenatal diagnosis, some major challenges have been actively debated as prenatal WES clinically is becoming part of routine testing. The lack of an accurate fetal phenotype achieved by ultrasonography, variable disease descriptions, and appearances of ultrasound findings further complicate the interpretation of the WES results (Best et al., 2018). Other challenges including the cost effectiveness of WES (Aaltio et al., 2022) and even social and ethical issues (Horn and Parker, 2018) are also taken into account. There are several limitations in the current study which need to be further improved and supplemented. First, we performed WES only for the proband instead of parental–fetal trios. Since trio-based WES testing would be helpful for variant filtering in initial data analysis and enable rapid identification of *de novo* variants. In particular, for WES negative cases, trio-based WES is useful for determining the causative genes. Thus, parental–fetal trio analysis is preferable, although it would increase the cost. Second, this is a retrospective study; some of the pregnancy outcomes were obtained through follow-up phone calls rather than confirmation of diagnosis by a clinician; thus, we are not sure whether the parents have given an objective statement of fact. Finally, the relatively small sample size is still a limitation to our study so that we are unable to identify statistical differences of diagnostic rates between different disorders. Therefore, additional efforts are urgent on large cohort studies to better clarify correlations between the fetal genotype and phenotype and provide refined prenatal counseling.

## 5 Conclusion

In conclusion, we confirmed the potential of WES to improve prenatal diagnosis in a cohort of 60 cases. Our results demonstrated that WES remarkably increased the prenatal diagnosis rate by 23.3% in fetuses with abnormal ultrasound findings but normal G-band karyotyping and CMA results. In addition, we suggested that WES may be recommended when conventional prenatal methods fail to provide a diagnosis in fetuses with ultrasonographic anomalies, particularly for disorders involving the skeletal system and head or neck systems. More importantly, this study identified five P/LP variants that had not been included in the HGMD. Additionally, three VUSes that had been reported at least once previously were recurred in this study. All of these findings further add to our current knowledge of phenotype–genotype relationships, which will improve the ability of genetic counselors to advice families.

## Data Availability

The datasets presented in this article are not readily available because of privacy restrictions. Requests to access the datasets should be directed to the authors.
