# Supplementary material for: Prenatal diagnosis of fetuses with ultrasound anomalies by whole-exome sequencing in Luoyang city, China
Source: Front Genet. 2024 Jan 22;14:1301439. doi: 10.3389/fgene.2023.1301439 (PMC10838985; doi:10.3389/fgene.2023.1301439)
Supplement: Supplementary file 1 [file Table2.DOCX]

**Supplementary Table S2 Secondary findings revealed by whole exome sequencing.**

| **Case** | Main ultrasound findings | Gene | Associated disorder^＃^ | Alteration | Variant type | HGMD  inclusion | Function prediction | Inheritance/ Zygosity | ACMG classification  (Green et al., 2013) | Pregnancy  outcome**^†^** |
| --- | --- | --- | --- | --- | --- | --- | --- | --- | --- | --- |
|  |  |  |  |  |  |  | REVEL/spliceAI effect |  |  |  |
| **27** | Polyhydramnios, bilateral femur asymmetry | *GJB2* | Epicophosis | NM_004004 exon2  c.109G>A  (p.V37I) | Missense | CM077559 | 0.656 | Inherited/ Homozygous | **Pathogenic**  (PS3_Supporting+PM3_Very Strong+PP1_Strong) | Live birth without  abnormity |
|  |  | *DUOX2* | Thyroid endocrine disorder | NM_014080 exon14  c.1588A>T  (p.K530*) | Nonsense | CM085369 | NA | Inherited/ Compound  heterozygous | **Pathogenic**  (PVS+PM3+PM2_Supporting) |  |
|  |  | *DUOX2* |  | NM_014080 exon9  c.971T>G  (p.I324S) | Missense | - | 0.809 |  | VUS  (PM3+PM2_Supporting+PP3) |  |
| **28** | Absence of the left kidney; single umbilical artery | *TTN* | Hypertrophic cardiomyopathy | NM_001267550 exon64  c.18696G>A  (p.W6232*) | Nonsense | CM086851 | NA | Inherited marternally/ Heterozygous | **Likely pathogenic**  (PVS+PM2_Supporting) | TOP |

“-” denotes the variant was not be included in HGMD;

“TOP” denotes termination of pregnancy;

“**†**” After termination, the fetal samples were not used for further diagnosis;

“＃”associated disorder was determined according to OMIM;

“NA” denotes not applicable.
