# Supplementary material for: Prenatal diagnosis of fetuses with ultrasound anomalies by whole-exome sequencing in Luoyang city, China
Source: Front Genet. 2024 Jan 22;14:1301439. doi: 10.3389/fgene.2023.1301439 (PMC10838985; doi:10.3389/fgene.2023.1301439)
Supplement: Supplementary file 2 [file Table3.DOCX]

**Supplementary Table S3 Summary of** **variants of uncertain significance revealed by whole exome sequencing.**

| **Case** | Main ultrasound findings | Gene | Associated disorder | Alteration | Variant type | HGMD  inclusion | Function prediction | Inheritance/ Zygosity | ACMG classification  (Green et al., 2013) | Pregnancy outcome**^†^** |
| --- | --- | --- | --- | --- | --- | --- | --- | --- | --- | --- |
|  |  |  |  |  |  |  | REVEL score |  |  |  |
| **29** | Unilateral microphthalmos | *OTX2* | Microphthalmia, syndromic 5 | NM_001270524.2  c.202C>T  (p.R68*) | Nonsense | - | NA | Inherited marternally/ Heterozygous | VUS  (PVS_Strong+PM2_Supporting) | TOP |
| **30** | Hypoplasia of fetal nasal bone | *TRPS1* | Trichorhinophalangeal syndrome, type I | NM_014112.5  c.3461A>G  (p.T1154C) | Missense | - | 0.690 | Inherited marternally/ Heterozygous | VUS  (PM2_Supporting+PP3) | Live birth without abnormity |
| **31** | Multiple pulmonary cysts | *JAG1* | Alagille syndrome 1 | NM_000214.3  c.1136C>T  (p.S379F) | Missense | CI992790 | 0.635 | Inherited paternally/ Heterozygous | VUS  (PM2_Supporting) | Live birth without abnormity |
|  |  | *GATA6* | Pancreatic agenesis and congenital heart defects | NM_005257.6  c.422C>A  (p.P141H) | Missense | - | 0.313 | Inherited paternally/ Heterozygous | VUS  (PM2_Supporting) |  |
| **32** | Atrioventricular canal defect | *KIF2A* | Cortical dysplasia, complex, with other brain malformations 3 | NM_001243952.2  c.1499A>G  (p.E500G) | Missense | - | 0.568 | De novo/ Heterozygous | VUS  (PM2_Supporting) | TOP |
| **33** | Intrauterine growth restriction,short fetal femur length | *NBAS* | Infantile liver failure syndrome 2 | NM_015909.4  c.6124A>G  (p.M2042V) | Missense | CM212137 | 0.037 | Inherited/ Compound  heterozygous | VUS  (PM2_Supporting) | Live birth without abnormity |
|  |  |  |  | NM_015909.4  c.3938C>T  (p.P1313L) | Missense | - | 0.083 |  | VUS  (PM2_Supporting) |  |

**Continued**

| **Case** | Main ultrasound findings | Gene | Associated disorder | Alteration | Variant type | HGMD  inclusion | Function prediction | Inheritance/ Zygosity | ACMG classification | Pregnancy outcome**^†^** |
| --- | --- | --- | --- | --- | --- | --- | --- | --- | --- | --- |
|  |  |  |  |  |  |  | REVEL/spliceAI effect |  |  |  |
| **34** | Short fetal femur and humerus length | *DYNC2H1* | Short-rib thoracic dysplasia 3 with or without polydactyly | NM_001080463.2  c.7409C>T  (p.A2470V) | Missense | CM182055 | 0.250 | Inherited/ Compound  heterozygous | VUS  (PM2_Supporting+PP3) | TOP |
|  |  |  |  | NM_001080463.2  c.880T>G  (p.W294G) | Missense | - | 0.732 |  | VUS  (PM2_Supporting+PP3) |  |
| **35** | Polycystic kidney dysplasia | *GANAB* | Polycystic kidney disease 3 | NM_001278192.2  c.232G>A  (p.V78I) | Missense | - | 0.148 | Inherited marternally/ Heterozygous | VUS  (PM2_Supporting) | Live birth without abnormity |
| **36** | Fetal ventriculomegaly | *VSX1* | Craniofacial anomalies and anterior segment dysgenesis syndrome | NM_001256271.2  c.570T>C  (p.D190D) | Synonymous | - | - | Inherited marternally/ Heterozygous | VUS  (PM2_Supporting) | Live birth with polymicrogyria |
| **37** | Short fetal femur and humerus length | *MCM5* | Meier-Gorlin syndrome 8 | NM_006739.4  c.1804C>T  (p.R602C) | Missense | - | 0.107 | Inherited/ Compound  heterozygous | VUS  (PM2_Supporting) | Moderate postnatal growth restriction |
|  |  |  |  | NM_006739.4  c.497C>T  (p.T166I) | Missense | - | 0.370 |  | VUS  (PM2_Supporting) |  |

**Continued**

| **Case** | Main ultrasound findings | Gene | Associated disorder^＃^ | Alteration | Variant type | HGMD  inclusion | Function prediction  REVEL/spliceAI effect | Inheritance/ Zygosity | ACMG classification | Pregnancy outcome**^†^** |
| --- | --- | --- | --- | --- | --- | --- | --- | --- | --- | --- |
| **38** | Osteogenesis Imperfecta | *FGFR3* | Hypochondroplasia | NM_000142.5  c.247C>T  (p.P83S) | Missense | - | 0.018 | Inherited paternally/ Heterozygous | VUS  (PM2_Supporting) | Live birth without abnormity |
|  |  | *TRPV4* | Spondylometaphyseal dysplasia, Kozlowski type | NM_021625.5  c.846C>G  (p.F282L) | Missense | - | 0.505 | Inherited marternally/ Heterozygous | VUS  (PM2_Supporting) |  |
| **39** | Short fetal femur and humerus length | *KIF22* | Spondyloepimetaphyseal dysplasia with joint laxity, type 2 | NM_007317.3  c.101G>C  (p.G34A) | Missense | - | 0.111 | Inherited paternally/ Heterozygous | VUS  (PM2_Supporting) | Loss to follow-up |
|  |  | *RNF125* | Tenorio syndrome | NM_017831.4  c.344A>G  (p.H115R) | Missense | - | 0.518 |  | VUS  (PM2_Supporting) |  |
| **40** | Fetal pyelectasis and abnormality of the ureter | *PTPN11* | LEOPARD syndrome 1 | NM_001330437.2  c.302C>G  (p.P101R) | Missense | - | 0.938 | Inherited marternally/ Heterozygous | VUS  (PM2_Supporting+PP3) | Live birth without other information |
|  |  | *PKD1* | Polycystic kidney disease 1 | NM_001009944.3  c.11659C>G  (p.P3887A) | Missense | - | 0.434 |  | VUS  (PM2_Supporting) |  |
| **41** | Bilateral talipes equinovarus | *GSC* | Short stature, auditory canal atresia, mandibular hypoplasia, skeletal abnormalities | NM_173849.3  c.499T>C  (p.F167L) | Missense | - | 0.948 | Inherited paternally/ Heterozygous | VUS  (PM2_Supporting+PP3) | Loss to follow-up |
| **42** | Bilateral talipes equinovarus | *SOX9* | Campomelic dysplasia | NM_000346.4  c.592A>T  (p.I198F) | Missense | - | 0.495 | Inherited marternally/ Heterozygous | VUS  (PM1+PM2_Supporting) | Live birth bilateral talipes equinovarus |
| **43** | Tetralogy of Fallot | *NOTCH2* | Hajdu-Cheney syndrome | NM_024408.4  c.6998C>T (p.A2333V) | Missense | - | 0.276 | Inherited paternally/ Heterozygous | VUS  (PM2_Supporting) | TOP |

“-” denotes the variant was not be included in HGMD;

“TOP” denotes termination of pregnancy;

“**†**” After termination, the fetal samples were not used for further diagnosis;

“＃”associated disorder was determined according to OMIM;

“NA” denotes not applicable.
