# Supplementary material for: Prenatal diagnosis of fetuses with ultrasound anomalies by whole-exome sequencing in Luoyang city, China
Source: Front Genet. 2024 Jan 22;14:1301439. doi: 10.3389/fgene.2023.1301439 (PMC10838985; doi:10.3389/fgene.2023.1301439)
Supplement: Supplementary file 4 [file Table4.DOCX]

**Supplementary Table S4** **Main ultrasound findings and pregnancy outcomes among 17 cases with negative whole exome sequencing results.**

| **Case** | Main ultrasound findings | Pregnancy outcome**^†^** | **Case** | Main ultrasound findings | Pregnancy outcome**^†^** |
| --- | --- | --- | --- | --- | --- |
| **44** | Bowing of long bones | Live birth without abnormity | **53** | Bilateral talipes equinovarus and pleural effusion | TOP |
| **45** | Increased nuchal translucency | Live birth without abnormity | **54** | Bilateral hyperechogenic kidneys | TOP |
| **46** | Abnormal heart morphology and atrial septal defect | Loss to follow-up | **55** | Anomalous hepatic venous drainage into the left atrium | Live birth without abnormity |
| **47** | Atrial septal defect and overriding aorta | Live birth without abnormity | **56** | Short femur | Live birth with slightly postnatal growth restriction |
| **48** | Bilateral talipes equinovarus | TOP | **57** | Skeletal dysplasia | TOP |
| **49** | Increased nuchal translucency | Live birth without abnormity | **58** | Hypoplasia involving bones of the lower limbs | Live birth without abnormity |
| **50** | Intrauterine growth retardation and atrial septal defect | Loss to follow-up | **59** | Abnormal heart morphology | Live birth without abnormity |
| **51** | Short femur | Live birth without abnormity | **60** | Hypoplastic facial bones | Live birth without abnormity |
| **52** | Intrauterine growth retardation | Live birth without abnormity |  |  |  |

“**†**” After termination, the fetal samples were not used for further diagnosis;

“TOP” denotes termination of pregnancy;
